# Nature imagery's influence on ERN amplitude: an examination of Attention Restoration Theory using EEG

**DOI:** 10.3389/fnhum.2025.1567689

**Published:** 2025-06-17

**Authors:** Sara A. Collins, Amy S. McDonnell, Emily E. Scott, Glen D. McNay, Mary F. Shannon, Lensky Augustin, Janet Nicole Hoffmann, Sharde Johnson, David L. Strayer, Sara B. LoTemplio

**Affiliations:** ^1^Department of Human Dimensions of Natural Resources, Colorado State University, Fort Collins, CO, United States; ^2^Department of Psychology, University of Utah, Salt Lake City, UT, United States; ^3^Department of Psychology, Vermont State University, Johnson, VT, United States; ^4^Parks, Recreation and Tourism Department, University of Utah, Salt Lake City, UT, United States

**Keywords:** Attention Restoration Theory, EEG, nature and health, ERN, nature images, virtual nature, nature and cognition, attention

## Abstract

Empirical research on the mental health and cognitive benefits of nature immersion has expanded significantly in recent decades, building support for Attention Restoration Theory. However, the field still faces interpretive challenges due to inconsistent definitions of ‘nature' (whether nature imagery, real-world nature immersion, or other forms) and varied methodologies, which collectively limit our understanding of the underlying mechanisms that potentially drive these benefits. Addressing some of these limitations, the current study investigated whether exposure to virtual nature imagery influences attention restoration, as measured by the amplitude of the error-related negativity (ERN), similarly to real-world nature. In a repeated-measures randomized control design, 63 participants completed the Eriksen Flanker Task at three testing sessions. At Session 1, participants completed the task after viewing a neutral stimulus for 10 minutes. At Session 2, participants completed the task after viewing either nature or urban imagery for 10 minutes. At Session 3, participants completed the task after viewing the neutral stimulus again for 10 minutes. The ERN component generated from the Eriksen Flanker Task was quantified at each of the three testing sessions to assess changes in cognitive control and error monitoring associated with viewing different types of environmental imagery. Results showed no significant differences in ERN amplitude across sessions or between nature imagery and urban imagery at Session 2. Collectively, these results suggest that brief exposure to the 2-D nature imagery used within this study may not elicit the same attention-dependent responses as real-world nature exposure.

## 1 Introduction

Research into the health benefits of nature exposure has grown substantially, with particular attention paid to its effects on mental health and cognitive function (Jimenez et al., 2021; Kuo, 2015; LoTemplio et al., 2023a). Two theoretical frameworks have emerged to explain the mechanistic pathways underlying these cognitive changes: Attention Restoration Theory (ART; Kaplan, 1995) and Stress Recovery Theory (SRT; Ulrich et al., 1991). SRT suggests that natural environments reduce mental stress and enhance recovery from stress compared to urban environments (Ulrich et al., 1991). Meanwhile ART suggests that natural environments enable cognitive recovery through passive attention to natural stimuli, whereas urban environments demand sustained attention and continuous inhibition of irrelevant stimuli, leading to cognitive fatigue and impaired restoration (Kaplan, 1995).

ARTS's foundation lies in distinguishing between two fundamental attentional systems: top-down and bottom-up processing. Top-down attention involves voluntary cognitive effort to focus on specific stimuli while suppressing others, whereas bottom-up attention represents an involuntary, effortless process triggered by salient environmental stimuli (Connor et al., 2004; Corbetta and Shulman, 2002; Katsuki and Constantinidis, 2014; for review see Petersen and Posner, 2012). ART proposes that natural environments engage primarily bottom-up processing (Buschman and Miller, 2007), while urban environments necessitate more demanding top-down processing, which can lead to cognitive depletion (Kaplan, 1995). This differential engagement predicts that nature exposure reduces cognitive fatigue, enhancing executive functioning and attentional resources, while urban exposure increases attentional demands and subsequent cognitive depletion.

Substantial evidence supports ART and nature's cognitive benefits (Kuo, 2015; LoTemplio et al., 2023a), with comprehensive reviews and meta-analyses demonstrating improved executive function, attention, and performance on cognitive tasks like backwards digit span in natural vs. urban environments (Jimenez et al., 2021; Ohly et al., 2016; Stenfors et al., 2019). Some studies even report declining performance after urban exposure, suggesting enhanced executive attention in natural settings. However, the underlying mechanisms of these effects remain poorly understood. The field faces challenges from inconsistent definitions of “nature” and attention assessements, impeding result interpretation and identification of which specific characteristics of attention are most influenced by nature exposure (Charbonneau et al., 2024; Ohly et al., 2016; Stevenson et al., 2018).

Another significant consideration in the current literature is the use of both real-world and nature imagery, with many studies relying on nature imagery to approximate the effects of real-world nature exposure (Johnson et al., 2021). Some studies show nature imagery successfully mimics real-world nature in improving cognitive performance (Berman et al., 2008; Crossan and Salmoni, 2021), as exemplified by Stevenson et al.'s (2018) finding of equivalent small yet significant effects on attention restoration between nature imagery and real-world nature exposures. However, these findings are not universal. A meta-analysis by Johnson et al. concluded that attentional control performance, a core component of executive attention, did not improve between a nature imagery or urban imagery intervention when measured by the Attention Network Task (2021; Fan et al., 2002). Yet contradictorily, Charbonneau et al. (2024) demonstrated that nature imagery *does* improve attentional control using a Flanker Task with an imposed deadline to avoid confounds with reaction time or speed accuracy tradeoffs (Draheim et al., 2021).

These contradictions and the lack of definitive understanding of mechanistic pathways underscore the critical need to develop a more nuanced understanding of nature imagery's impact on specific attributes of cognition and attention.

Given the significant heterogeneity in behavioral results of nature on attention, neuroscience methodologies could provide a useful converging method to disentangle which aspects of attention are specifically influenced by nature, and under which circumstances (Jimenez et al., 2021; Ohly et al., 2016). Neurophysiological measures, with their high sensitivity to subtle neural changes that often do not manifest in overt behavioral changes, offer the opportunity to further illuminate unique insights into the underlying components of attention that are influenced by distinct types of nature. Despite their promising role in this field, limited research has included neurological components of measurements in their assessments.

Nature and health research has leveraged Functional Magnetic Resonance Imaging (fMRI) to examine how natural stimuli influence cognitive processing through blood oxygen level-dependent signals (Ekstrom, 2010; Ogawa et al., 1992). Urban scenes have been shown to elicit increased activity in the occipital lobe and posterior cingulate cortex, regions associated with effortful visual processing and voluntary attention allocation (Hasler et al., 2007; Hölzel et al., 2011; Jiang et al., 2023; Joye et al., 2024; Kim et al., 2010; LeDoux, 2000; Norwood et al., 2019; Veer et al., 2011). In contrast, natural scenes preferentially activate areas linked to involuntary attention and sensory processing, particularly the inferior frontal gyrus and parietal regions (Jiang et al., 2023; Kim et al., 2010; Luo, 2018; Scott et al., 2023; Wang et al., 2019). These differential activation patterns may provide neurobiological support for Attention Restoration Theory, demonstrating that nature scenes engage less cognitively demanding neural pathways compared to urban environments (Norwood et al., 2019).

While fMRI has suggested functional differences between nature and urban imagery, its limited temporal resolution constrains the ability to link specific neural activation patterns to precise cognitive processes in response to stimuli. This temporal constraint, combined with its cost and immobility, makes it difficult to establish direct relationships between neural activation and cognitive improvements in attention in nature. Electroencephalography (EEG) addresses these limitations through high temporal resolution, direct neural activity measurement, cost-effectiveness, and portability (Scott et al., 2021), enabling more precise investigation of how natural vs. urban environments influence attention and executive function over time.

Early investigations of nature's neural impact through EEG focused on alpha wave activity. Ulrich's (1983) pioneering work revealed increased alpha wave activity during nature compared to urban imagery viewing, a finding later corroborated by Chang et al. (2008). While increased alpha wave activity was initially interpreted as indicating decreased wakefulness and attentiveness, subsequent research has revealed a more complex relationship between alpha activity and attention in natural environments. Grassini et al. (2019) suggest that increased alpha waves indicate that natural environments may stimulate restorative processes involved in cognition and emotion. Conversely, Hopman et al. (2020) found *decreased* resting posterior alpha (PA) during a 4-day nature immersion, suggesting enhanced external attentional focus rather than internal processing. These contradictory findings likely reflect different operational definitions of alpha activity and varying exposure contexts (e.g., viewing nature imagery vs. immersion in real nature).

More recent research has examined frontal theta waves, neural oscillations linked to attentional effort that reliably increase with heightened attentional demands (Chikhi et al., 2022). McDonnell and Strayer (2024a) quantified frontal theta waves at rest (i.e., not during a cognitive task) before and after a comparable 40-min nature vs. urban walk, finding significant increases after urban walks but not nature walks. This suggests urban environments place high demands on attentional systems while natural environments allow attentional rest, potentially enabling enhanced performance when cognitive engagement resumes.

These diverse EEG findings demonstrate natural stimuli's unique impact attention systems. Though their interpretation in relation to ART varies. The seemingly contradictory results suggest that the relationship between natural environments and attention involves multiple neural mechanisms operating at different temporal scales, underscoring the need for more precise temporal measurements to understand these complex cognitive processes. Additionally, oscillatory activity (i.e., alpha and theta waves) is typically recorded at rest, losing complex dynamics of task-related attentional control in nature vs. urban environments.

Event-Related Potentials (ERPs) provide precise temporal measurements of neural responses to specific events, offering critical insights into task-related cognitive processing. These components are particularly valuable in understanding how the brain processes natural vs. urban environments, and how the brain's attentional control processes change in real-time as a result of immersion in nature or urban environments. Indeed, ERP studies demonstrate differential neural responses to natural vs. built environments. During nature viewing, studies examining various ERP components such as the N1 (associated with early allocation of attentional resources and prioritization of relevant sensory information; Näätänen and Michie, 1979), EPN (reflecting arousal, motivation, and emotional responses influencing selective attention; for review, see Schupp et al., 2006), and P3 (involved in higher-order cognitive processes including selective attention, stimulus evaluation, and attentional resource allocation, often linked to novel stimulus detection; for review, see Friedman et al., 2001) reveal distinct neural mechanisms underlying attention and cognition. Together, these findings suggest that nature viewing reduces attentional processing loads relative to urban image viewing, as demonstrated by reduced ERP amplitudes in nature contexts (Grassini et al., 2019). This aligns with ART, which posits that natural environments promote attentional recovery by downregulating attention networks at rest.

The Error-Related Negativity (ERN) component has emerged as an empirically valid and informative ERP used for assessing attention and cognitive control during a task (Gehring et al., 1993, 2012). The ERN, a time-fixed negative deflection waveform reflecting the difference between error and correct response trials (LoTemplio et al., 2020), is thought to be generated by the anterior cingulate cortex (ACC; LoTemplio et al., 2020; Posner and Dehaene, 1994; for review see LoTemplio et al., 2023). The ACC is thought to allocate cognitive control resources in response to changing environmental demands (Shenhav et al., 2013) and operates within the executive attention network to regulate error monitoring and cognitive resource allocation (Geva et al., 2013; Van Steenbergen and Band, 2013).

The ERN is particularly well-suited for assessing attention restoration as the ERN's amplitude has been consistently linked to attention allocation, goal-maintenance, and cognitive control processes, with larger (more negative) ERN amplitudes corresponding to greater attentional control resources available (Gehring et al., 2012; LoTemplio et al., 2020; Luck and Kappenman, 2013; see LoTemplio et al., 2023a for review). The ERN amplitude serves as an ideal neural marker for testing ART's prediction about nature restoring depleted attention due to its direct relationship with attentional resource availability. While behavioral measures can be influenced by multiple cognitive processes, the ERN provides a specific neural signature of error-monitoring processes tied directly to executive attention. This allows for more precise measurement of the attentional restoration effects predicted by ART. The ERN's sensitivity to subtle changes in attentional resources enables detection of effects that might not be captured by behavioral measures alone, thus addressing methodological limitations identified in previous nature exposure research (McDonnell and Strayer, 2024b; LoTemplio et al., 2020).

Recent empirical evidence has established the ERN's validity as a measure of attention restoration specifically in nature contexts. LoTemplio et al. (2020) investigated ERN responses during the Eriksen Flanker Task (Eriksen and Eriksen, 1974) across a 5-day wilderness excursion. Their findings revealed increased ERN amplitude following nature exposure, a result recently replicated by McDonnell and Strayer (2024b), who demonstrated enhanced ERN amplitude after a 40-min nature walk but not after an equivalent urban walk. Additionally, McDonnell and Strayer (2024b) found that as perceived restorativeness of nature walks increased, so did ERN amplitude, establishing a direct link between subjective restoration experiences and this neural marker.

These findings align with ART's framework: while natural environments enable attentional networks to rest during exposure, they simultaneously restore attentional resources that become available for subsequent cognitive demands. As the ERN reflects capacity of executive function, its increased amplitude during the cognitively demanding Flanker task suggests that participants who experienced nature-induced restoration had more attentional resources available to allocate to the task at hand. This enhanced capacity for executive function manifests as higher ERN amplitudes during task performance, indicating more robust error monitoring and cognitive control processes (LoTemplio et al., 2020; McDonnell and Strayer, 2024b).

However, a critical discrepancy exists in the literature regarding the effectiveness of nature imagery. Johnson et al.'s (2021) meta-analysis found no significant effect of nature imagery on behavioral measures of attention restoration and executive function. However, neurophysiological studies suggest that natural stimuli do influence underlying cognitive processes in ways that may not be captured by behavioral measures alone. The exceptional sensitivity of ERP measurements can detect subtle shifts in attention and cognitive processing that behavioral tasks might miss.

Therefore, the use of EEG can assist in more deeply understanding nature's impact on attention restoration and cognition. Additionally, much of the current literature on nature and health relies on nature imagery in assessing nature's impact on health. Johnson et al. (2021) suggests that real-world nature may be more effective in eliciting attention restoration than nature imagery. However, as mentioned, it is possible that images produce effects on attention that may be present in the brain but not in behavior. As the ERN has been demonstrated to be significantly impacted by nature exposure and is a reliable measure of executive function, it would serve as an excellent tool to address this gap in our understanding. Understanding the specific neural mechanisms of the “nature effect” on attention could help clarify which environmental characteristics confer specific attentional benefits. While Johnson et al. (2021) suggests that real-world nature may be more effective than nature imagery for attention restoration, no research has yet investigated nature imagery's impact on ERN amplitude. Such investigation could help resolve current discrepancies between behavioral and neural findings while deepening our understanding of nature's impact on attention restoration.

The present study uses EEG to examine the ERN response while performing the Eriksen Flanker Task at three distinct testing sessions. At Session 1, participants completed the task after viewing a neutral stimulus for 10 min. At Session 2, participants completed the task after viewing either nature or urban imagery for 10 min. At Session 3, participants completed the task after viewing the neutral stimulus again for 10 min. The current study's purpose is to determine if exposure to nature imagery (at Session 2) influences the amplitude of the ERN similarly to what has been demonstrated in real-world nature (LoTemplio et al., 2020; McDonnell and Strayer, 2024b). Based on ART and previous findings demonstrating enhanced error monitoring following nature exposure (LoTemplio et al., 2020; McDonnell and Strayer, 2024b), we hypothesized that ERN amplitude would significantly increase after viewing nature imagery but not urban imagery, reflecting greater availability of attentional resources for cognitive control processes specifically following nature exposure.

## 2 Material and methods

### 2.1 Study design

**Figure 1:**
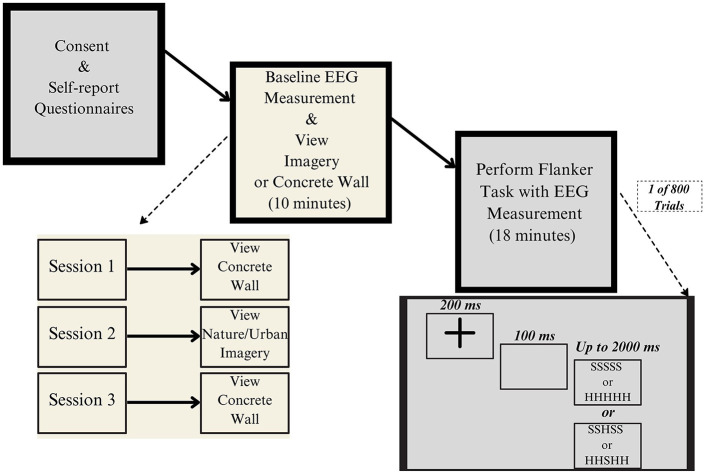
Flow chart illustrating the overall study design, with the start of the process on the left and the end on the right. A dotted arrow indicates the individual session design, specifying whether participants viewed the concrete wall (i.e., a neutral stimulus) or the nature/urban image condition.

The study employed a 2 (environmental manipulation: nature vs. urban) × 3 (time) between-subjects repeated-measures design, with participants completing three 2-h sessions over a 3-week period (see Figure 1 for overview of overall study design). Prior to each session, participants completed standardized questionnaires assessing potential physiological confounds: 24-h alcohol consumption (quantified as number of drinks), caffeine intake (relative to typical daily consumption), and sleep duration (relative to typical patterns; see Table 1).

**Table 1:** Response to questionnaires assessing potential physiological confounds.

**Measure**	**Session 1**	**Session 2**	**Session 3**
**Alcohol**	**(*****N*** = ***54*****)**	**(*****N*** = ***58*****)**	**(*****N*** = ***55*****)**
None	44	46	47
Low	9	10	7
High	1	1	0
N/A	0	1	1
**Sleep**	**(*****N*** = ***54*****)**	**(*****N*** = ***58*****)**	**(*****N*** = ***55*****)**
No	5	9	7
Yes	49	49	47
N/A	0	0	1
**Caffeine**	**(*****N*** = ***54*****)**	**(*****N*** = ***58*****)**	**(*****N*** = ***55*****)**
No	10	10	11
Yes	44	48	44

Alcohol consumption categorized as: None = 0 drinks, Low = 1–3 drinks, High = 4–7 drinks within previous 24 h. Caffeine intake reported dichotomously (yes/no) relative to participant's typical daily consumption, where “yes” indicates typical consumption. Sleep adequacy reported dichotomously (yes/no) relative to participant's typical sleep pattern, where “yes” indicates typical duration. N/A indicates that a participant did not respond to the given question. Values represent frequency of participants in each category per session.

**Figure 2:**
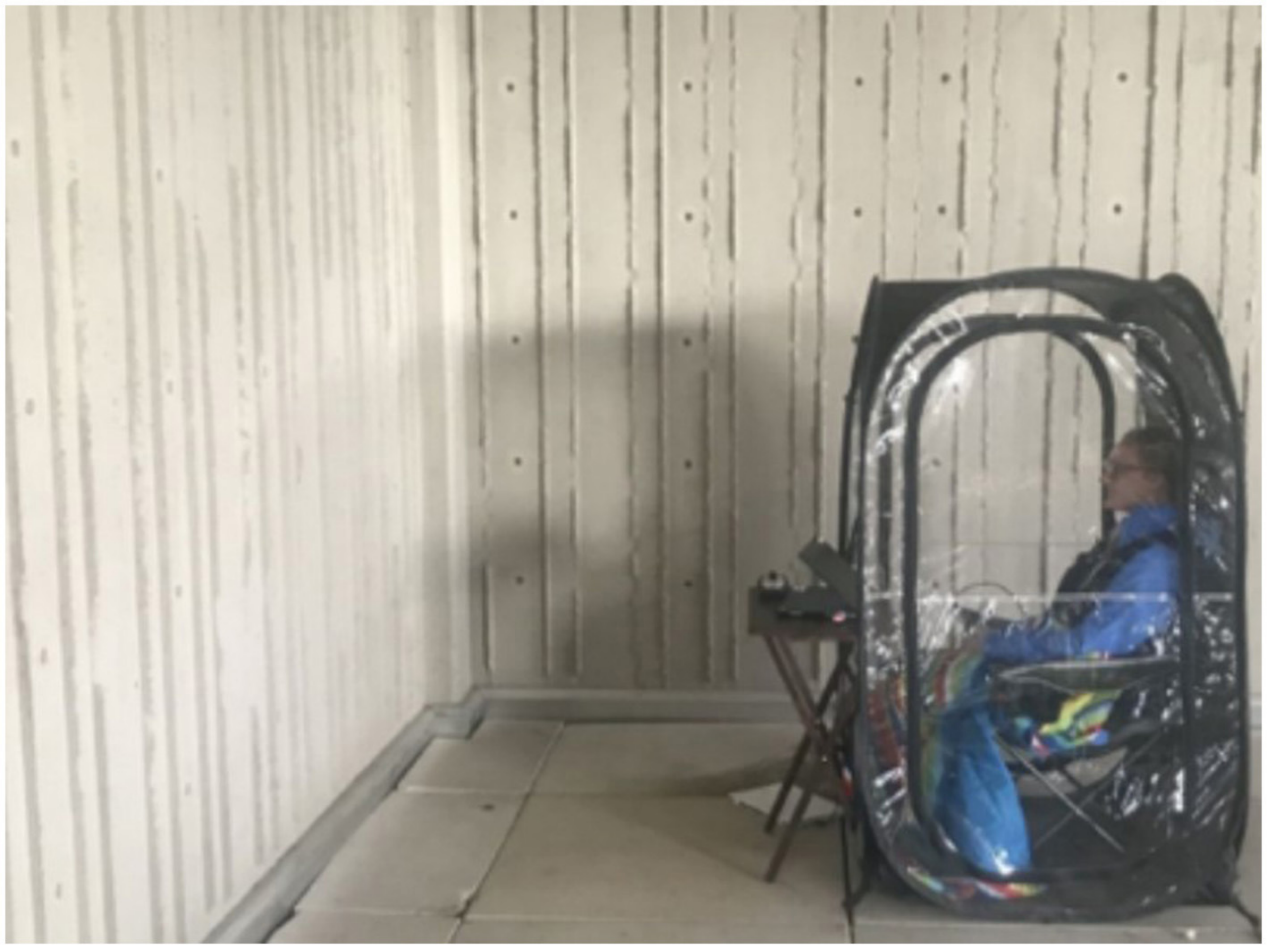
Illustration of outdoor recording set up used in all three testing sessions.

**Figure 3:**
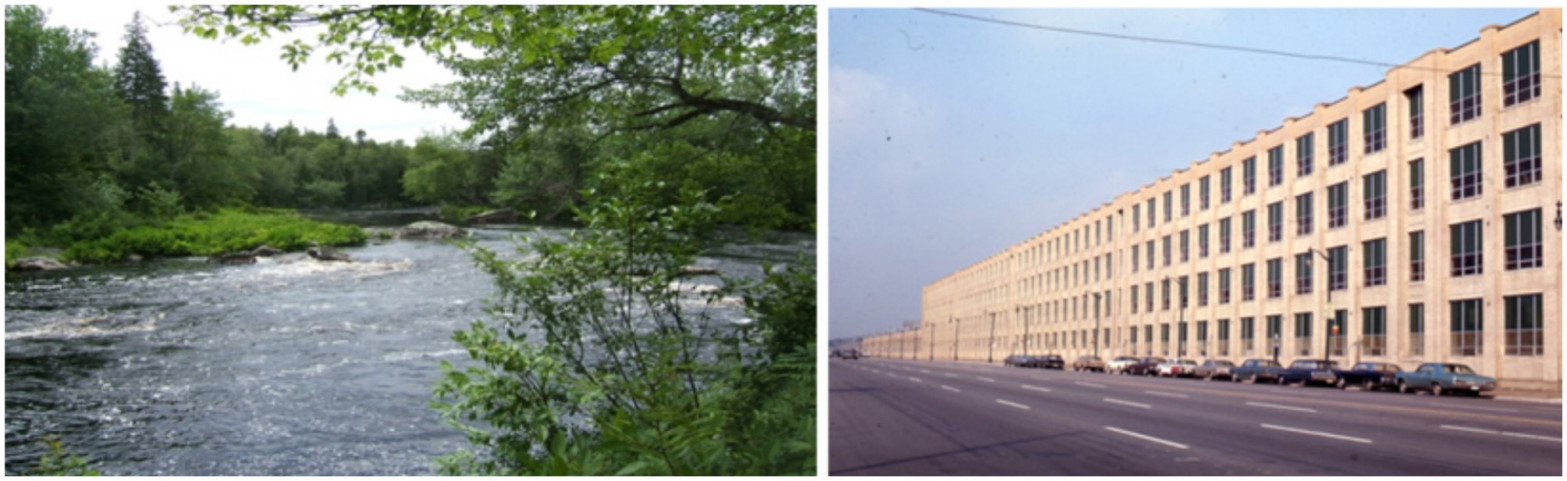
Example of nature **(left)** and urban **(right)** images used at session 2.

During Session 1 (the baseline session) and Session 3 (the post-intervention session), participants viewed a neutral concrete wall for 10 min at a designated outdoor location adjacent to the laboratory (Figure 2). For the experimental manipulation (Session 2), participants returned to the identical location and were randomly assigned to view either urban or nature imagery for 10 min (Figure 3). This design and length time for imagery exposure replicated established environmental exposure protocols (LoTemplio et al., 2020) while maintaining consistent EEG recording conditions across sessions. The use of a neutral viewing condition in Sessions 1 and 3 enabled isolation of the imagery manipulation effects on ERN amplitude by controlling for potential environmental confounds.

After viewing, participants completed a high-congruency version of the Flanker Task (Eriksen and Eriksen, 1974) programmed in E-Prime 2.0 while EEG was recorded. The task presented a horizontal array of five letters, with participants responding to the central target letter across all sessions. Stimuli were either congruent (e.g., SSSSS or HHHHH) or incongruent (e.g., SSHSS or HHSHH), where incongruent trials featured flanking letters associated with the opposite response (Figure 1). Each trial began with a central fixation cross displayed for 200 ms, followed by a 100-ms blank screen. The stimulus then appeared and remained visible until either the participant responded, or 2,000 ms elapsed. Participants performed two blocks of 400 trails of the Flanker Task for a total of 800 trials.

### 2.2 Participants

Sixty-three participants (age range 18–40 years) from the greater Salt Lake City community were recruited. All participants had normal neurological functioning and normal or corrected-to-normal vision. Participants received $70 compensation for their participation. Three participants were excluded due to inadequate quality EEG data that resulted in 2 or more sessions' data missing. A final sample of 60 participants (*N* = 60; demographic details in Table 2) was included. Sensitivity analysis conducted using PANGEA (Westfall, 2016) confirmed that this sample size provided sufficient power (0.80, alpha = 0.05) to detect a medium effect size of *f* = 0.29.

**Table 2:** Self-reported demographics of participants.

**Variable**	**Frequency**	**Percentage**
**Age**	**(*****N*** = **60)**	**(** ***%*** **)**
18–24	35	58.33
25–34	21	35.00
35–44	4	6.66
**Gender/sex**	**(*****N*** = **60)**	**(** ***%*** **)**
Male	16	26.66
Female	44	73.33
Non-binary	0	0
Transgender	0	0
**Race**	**(*****N*** = **60)**	**(** ***%*** **)**
White	47	78.33
Asian	9	15.00
Black	1	1.66
Hispanic	1	1.66
White & Asian	2	3.33
**Handedness**	**(*****N*** = ***60*****)**	**(** ***%*** **)**
Left	2	3.33
Right	56	93.33
Ambidextrous	2	3.33

Participant Demographic Data.

Additionally, within individual testing sessions, some data points were excluded during processing, although the participants remained in the overall study. For Session 1, data from individual sessions was excluded for six participants: three had poor quality EEG files (e.g., excessive noise), while three performed the Flanker Task incorrectly with excessive errors (>500) due to misunderstanding instructions, resulting in unreliable ERN responses. In Session 2, data was excluded for two participants' sessions due to incorrect EEG recording that prevented ERN extraction. In Session 3, five participants' session data was excluded: three due to participant absence and two for insufficient error trials (<7; Meyer et al., 2013) after artifact correction for reliable ERN averaging.

### 2.3 Image presentation

The nature and urban images utilized in Session 2 were taken from Berman et al. (2008; see Figure 3 for example images). The images were presented via an ACER laptop and Microsoft Office PowerPoint. Each image was presented in a randomized order for 7 s and participants viewed the image sets for a total of 10 min (Berman et al., 2008; Berto, 2005). The laptop was placed about 24 inches from the participants' eyes, and they were instructed to passively watch the slideshow of images for the entire 10-min period prior to completing the Flanker Task.

### 2.4 EEG recording and processing

Data was collected using a BIOPAC system with gel-based passive electrodes (BIOPAC Systems, Goleta, CA, USA). Following the 10–20 system (Jasper, 1958), three scalp electrodes were placed at Fz, Cz, and Pz positions. A reference electrode was positioned on the right mastoid bone, and a ground electrode was placed on the forehead. Two additional electrodes were positioned above and below the right eye to record ocular movement artifacts, creating a bipolar vertical electrooculogram (VEOG) channel for artifact rejection. Electrode sites were prepared by light abrasion with NuPrep abrasive gel using a cotton swab, followed by electrode attachment using Ten20 conductive adhesive gel. Electrode impedances were maintained below 10 kΩ during recording, as verified by BIOPAC's EL-CHECK impedance checker. The electrode placement was designed to avoid interference with participants' field of view and range of motion. EEG data were acquired using BIOPAC Smart Center software (BIOPAC Systems, Goleta, CA, USA) with two wireless EEG transmitters. The wireless BioNomadix Smart Center amplified the EEG signal with a maximum sampling rate of 2,000 Hz per channel, and data were monitored online through AcqKnowledge software (Version 5.0). The BioNomadix Smart Center, a compact data acquisition unit and wireless receiver, connected to a computer's USB port and recorded simultaneous physiological data from each transmitter. No online filters were applied to the data. For communication between the E-Prime computer and EEG system, a USB-TTL Module (Black Box) was employed instead of a serial-to-DB9 converter cord, due to its enhanced compatibility with the EEG software.

The data was downsampled to a sampling rate of 250 Hz and subjected to a band-pass filter with a Butterworth filter type, from 0.1 to 30 Hz and a roll-off of 12 dB per octave. Data were epoched from −200 to 300 ms relative to response onset, with a −200 to −100 baseline period to avoid base lining into response activity that occurs *as* the participant is pressing the button. Time window selection was determined based on methods outlined in previous literature (LoTemplio et al., 2020). Gratton et al.'s (1983) regression method of eye movement correction was used to correct for vertical eye movements. Following EEG processing techniques outlined by LoTemplio et al. (2020), data was then viewed through moving-window artifact rejection function in ERPlab within EEGLAB in Matlab to correct for blinks that may have missed by the algorithm. Epochs were rejected if the VEOG channel's data deflected more than 100 μV within a 20 ms time period. Participants' data were removed if fewer than 7 error trials survived artifact correction (Meyer et al., 2013). Artifact-free ERPs were computed through response-locked averaging by response type (correct vs. error) after subtraction of the −200 to −100 ms pre-response baseline for the ERN. From these waveforms, difference waveforms (error-correct) were created for each subject and for each session (3 total per participant). The mean number of error trials included per session was *M* = 59.63 (*SD* = 38.77) for Session 1, *M* = 61.72 (*SD* = 47.94) for Session 2, and *M* = 64.37 (*SD* = 59.96) for Session 3. Mean amplitude of the difference waves was calculated using a 15–65 ms post-response window for all experimental conditions for each subject at electrode Cz. This electrode was chosen based on past literature that determined that the ERN was maximal at Cz (see LoTemplio et al., 2020).

### 2.5 Statistical analyses

#### 2.5.1 The amplitude of the error related negativity

Statistical analyses were performed in R (version 4.3.1). A linear mixed-effects model (LMM) was implemented using the *lmer* function from the *lme4* package (Bates et al., 2015) to evaluate the effects of session (1, 2, and 3) and image type (nature images vs. urban images) on the amplitude of the ERN. Significance testing using the *mixed* function from the *afex* package (Singmann et al., 2023) with the likelihood ratio test (LRT) method was used to generate omnibus main effects and interactions. An LMM was selected due to its robustness to moderate violations of residual normality and homoscedasticity (Schielzeth et al., 2020), its flexibility for modeling repeated measures, and its ability to incorporate random effects that account for subject-level variability, which can otherwise bias fixed-effect estimates. This approach also offers greater flexibility in handling unbalanced data, such as differences in the number of error trials across participants (Barr et al., 2013; Gelman and Hill, 2006; Luke, 2017; Schielzeth et al., 2020). The model included ERN amplitude as the dependent variable. Session, setting, and the interaction between session and setting were included as fixed effects and subject as a random effect within the model. Model diagnostics confirmed that residuals were approximately normally distributed (Shapiro-Wilk test, *p* > 0.05) and homoscedastic based on residual- vs.-fitted plots. Residuals appeared independent, with no systematic variation across session or condition. While Cook's distance identified several influential observations, these data points were retained after confirming from session notes that they reflected genuine variability rather than procedural anomalies.

#### 2.5.2 Behavioral data

Statistical analyses to evaluate the effects of session and setting on Flanker Task performance metrics were conducted following the same analytical framework used for the ERN amplitude analyses. Two separate LMMs were constructed: one with mean reaction time (RT) in milliseconds as the dependent variable, and another with mean accuracy (proportion correct) as the dependent variable. Both models incorporated session, setting, and their interaction as fixed effects, with subject as the random intercept. Trials with sub 200 ms responses were excluded as physiologically implausible.

Model assumptions for the reaction time model were evaluated prior to analysis. Histogram inspection indicated that residuals were approximately normally distributed, though a subtle bimodal pattern suggested mild deviation from a Gaussian distribution. The Shapiro-Wilk test was statistically significant, likely reflecting the test's sensitivity to minor deviations in larger samples rather than meaningful non-normality. Homoscedasticity was acceptable, with residuals evenly distributed across fitted values and conditions. Residuals appeared independent, with no systematic trends across session. Influential observations identified via Cook's distance were retained, as prior data cleaning had removed implausible values, and session documentation indicated no anomalies for high-leverage participants. These cases were interpreted as reflecting valid inter-individual variability.

Model assumptions for the accuracy model underwent parallel evaluation. Residuals exhibited significant non-normality (Shapiro-Wilk *W* = 0.9546, *p* < 0.001), with histogram inspection revealing clustering near the upper bound—indicative of a strong ceiling effect. Overall mean accuracy was 0.89, with narrow variation across conditions (0.88–0.90). Logit and arcsine square root transformations were applied but failed to normalize the residuals due to persistent clustering near 1, driven by the bounded nature of the data. Mild heteroscedasticity was observed, primarily compression of residuals near ceiling, but variance remained stable across conditions. Residuals appeared independent, and no systematic trends across session were detected. Influential observations were again retained based on thorough pre-modeling data cleaning and the absence of procedural issues. Given the established robustness of LMMs to moderate assumption violations (Schielzeth et al., 2020; Gelman and Hill, 2006), and the demonstrated reliability of the afex framework under conditions in which there are assumption violations (Barr et al., 2013; Luke, 2017), we proceeded with the planned analyses while interpreting results with appropriate caution.

Exploratory models were also run to confirm the presence of the expected Flanker effect. These included congruency as a fixed effect and subject as a random intercept. As these models were not central to our hypotheses, full assumption diagnostics are not reported here. However, residuals were visually inspected and did not indicate violations likely to substantially affect interpretation.

#### 2.5.3 Exploratory analysis: relationship between ERN and behavioral performance

To assess brain-behavior coupling in the context of attention restoration, we conducted an exploratory set of LMMs examining whether ERN amplitude predicted behavioral performance. These analyses clarify if neural and behavioral measures reflect distinct or overlapping measurements of attention restoration processes following nature exposure.

Four models were run: one predicting RT from ERN amplitude, one examining the interaction between ERN and session number on RT, and two identical models that predict accuracy rather than reaction time. All models included a random intercept for subject to account for repeated measures. We conducted assumption checks for all models following the same diagnostic approach described previously. No violations were found that would alter the interpretation of our findings.

## 3 Results

### 3.1 The amplitude of the error related negativity

Linear mixed effects models revealed no significant main effects of setting [$\chi_{\left(1\right)}^{2}$ = 0.25, *p* = 0.62], session [$\chi_{\left(2\right)}^{2}$ = 3.14, *p* = 0.21], or interaction between the two on ERN amplitude [$\chi_{\left(2\right)}^{2}$ = 2.05, *p* = 0.36].

**Figure 4:**
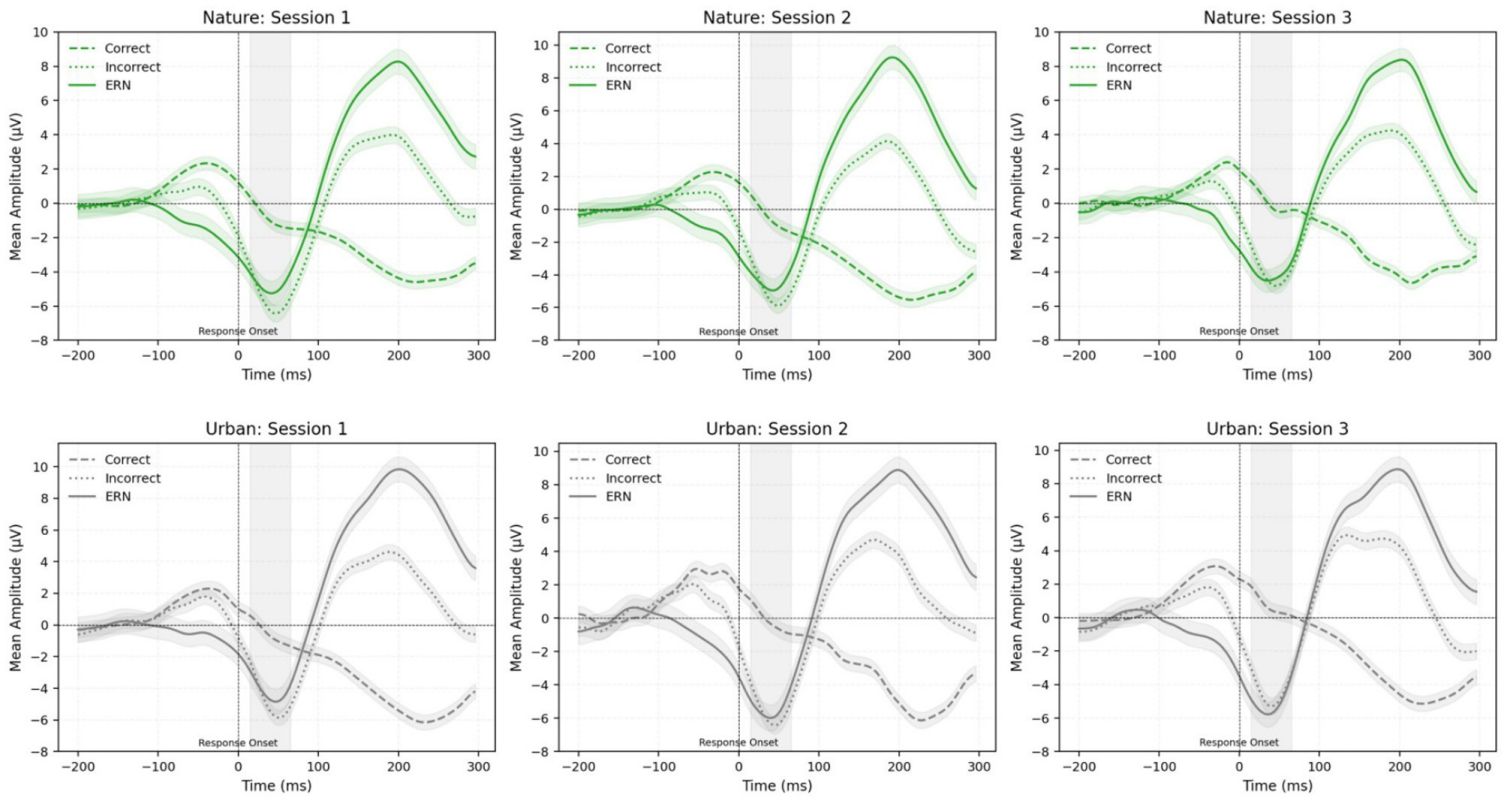
Mean amplitude of correct, incorrect, and ERN (incorrect minus correct) waveforms at electrode Cz, comparing sessions (1, 2, and 3) and image conditions (nature vs. urban). Green indicates the mean amplitude during the nature imagery condition and gray represents the mean amplitude during the urban imagery condition. Different line types (as shown in the top left corner of each panel) indicate correct, incorrect, and ERN trials across sessions and conditions. The gray shaded box (15–65 ms post-response) indicates the measurement window used for ERN extraction. Shaded areas surrounding each waveform represent the standard error of the mean.

**Figure 5:**
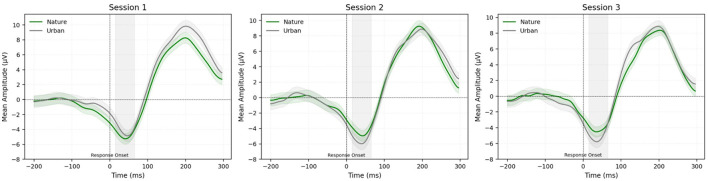
Grand average ERN (incorrect minus correct) mean amplitude at electrode Cz, comparing sessions (1, 2, and 3) and image conditions (nature vs. urban). Green waveforms represent the nature condition and gray waveforms represent the urban condition. The gray shaded box (15–65 ms post-response) indicates the measurement window used for ERN extraction. Shaded areas surrounding each waveform represent the standard error of the mean.

Figure 4 depicts the grand-averaged ERN waveforms at the Cz electrode site, comparing correct and incorrect response trials across all three sessions and across both image conditions. The ERN is represented as the difference in amplitude between correct and incorrect trials, with more negative values indicating larger ERN response. Figure 5 displays the grand average difference waveforms (calculated by subtracting correct from incorrect trial responses) at the Cz electrode, allowing for comparison of the ERN across settings (nature vs. urban) and sessions (see Table 3 for descriptive statistics).

**Table 3:** Descriptive statistics for mean ERN amplitudes (μV) across setting and sessions.

**Session**	**Setting**	***N***	***M***	***SD***
Session 1	Nature	26	−3.52	2.53
Session 1	Urban	28	−3.02	2.37
Session 2	Nature	28	−3.02	2.49
Session 2	Urban	30	−3.62	3.71
Session 3	Nature	27	−2.56	3.25
Session 3	Urban	28	−3.29	3.73

Negative values negative deflection of the ERN amplitudes, with more negative values representing stronger negative deflection. M, mean; SD, standard deviation.

### 3.2 Behavioral data

Congruency effects were evaluated for both performance metrics, with effect sizes quantified using Cohen's *d*. A significant Flanker effect emerged in behavioral measures, with faster reaction times [*t*_(271)_ = −20.27, *p* < 0.0001, *d* = 0.92] and higher accuracy [*t*_(271)_ = −21.20, *p* < 0.0001, *d* = 1.18] for congruent stimuli (i.e., HHHHH or SSSSS) compared to incongruent stimuli (i.e., SSHSS or HHSHH; see Table 4 for descriptive statistics).

**Table 4:** Descriptive statistics for congruent and incongruent stimuli.

**Stimulus type**	**M RT**	**SE RT**	**M accuracy**	**SE accuracy**
Congruent	457	7.59	0.95	<0.01
Incongruent	522	7.59	0.84	<0.01

RT indicates Reaction time in milliseconds and accuracy indicates proportion of correct responses. M, mean; SE, Standard Error.

Analysis of mean accuracy showed no significant main effects for setting [$\chi_{\left(1\right)}^{2}$ = 0.20, *p* = 0.65], session [$\chi_{\left(2\right)}^{2}$ = 0.16, *p* = 0.92], nor their interaction [$\chi_{\left(2\right)}^{2}$ = 0.05, *p* = 0.97]. The analysis of mean reaction time revealed no significant effect of setting [$\chi_{\left(1\right)}^{2}$ = 0.12, *p* = 0.72] but did show significant differences across sessions [$\chi_{\left(2\right)}^{2}$ = 29.68, *p* < 0.001]. Null results were found for the interaction between setting and session [$\chi_{\left(2\right)}^{2}$ = 1.09, *p* = 0.58]. An overview of model estimates is presented in Table 5.

**Table 5:** Model results for behavioral measures.

**Accuracy fixed effects**	***df***	**χ^2^**	***p***
Setting	1	0.20	0.65
Session	2	0.16	0.92
Setting^*^Session	2	0.05	0.97
**RT fixed effects**	***df***	*χ^2^*	***p***
Setting	1	0.12	0.72
Session	2	29.68	<0.001^***^
Setting^*^Session	2	1.09	0.58

^***^p <0.001, ^**^p <0.01, ^*^p <0.05.

Pairwise comparisons conducted using estimated marginal means with Tukey's correction for multiple comparisons (*emmeans* package; Lenth, 2025), revealed that reaction times differed significantly between Session 1 and Session 2 [*t*_(271)_ = 3.74, *p* < 0.001, Cohen's *d* = 0.32], and between Session 1 and Session 3 [*t*_(272)_ = 5.45, *p* < 0.0001, Cohen's *d* = 0.48], consistent with practice effects. No significant difference emerged between Sessions 2 and 3 [*t*_(272)_ = 1.86, *p* = 0.15].

**Figure 6:**
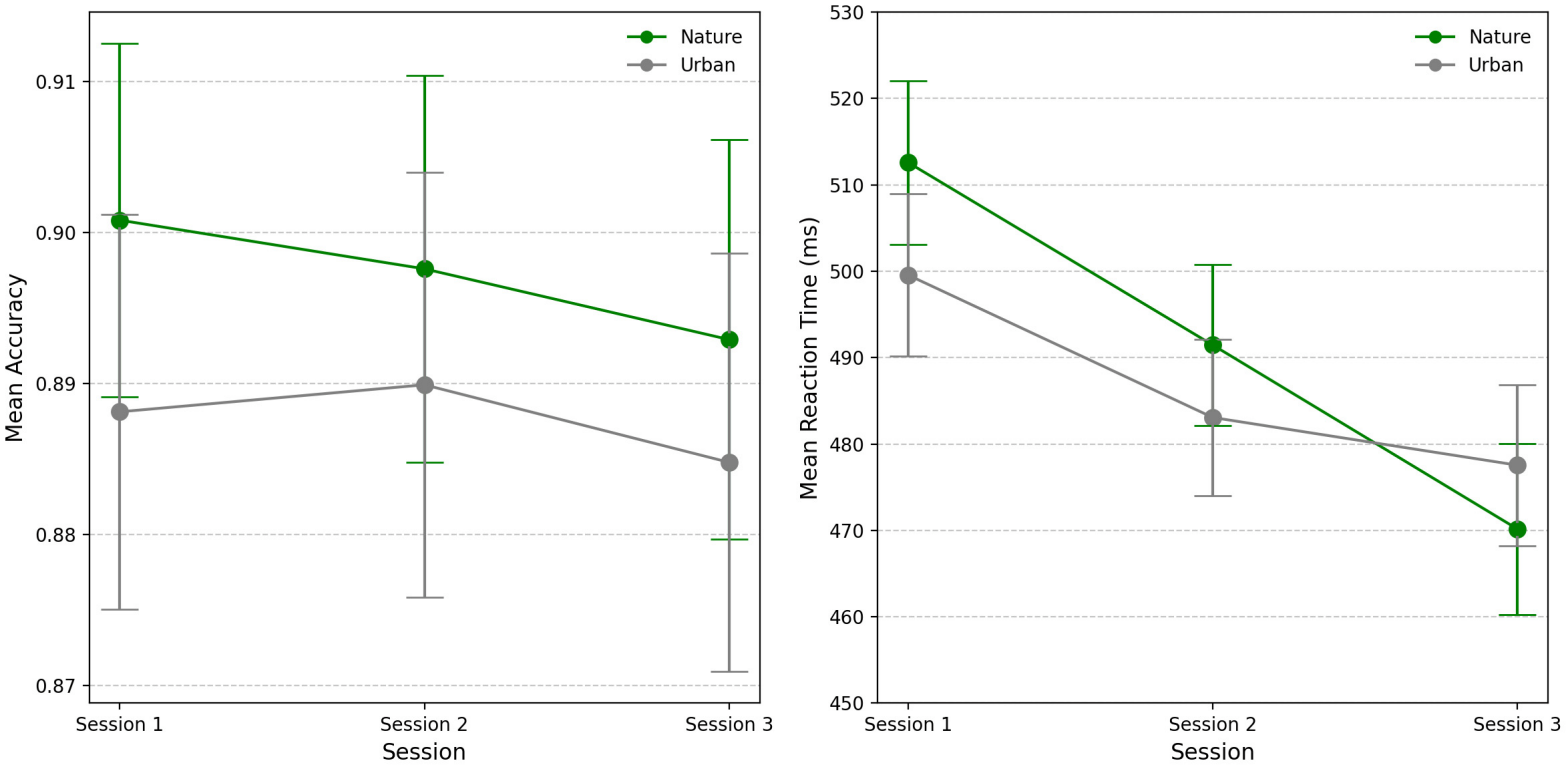
Observed means for accuracy (proportion of correct responses) and reaction time across session (1, 2, and 3) and condition (nature vs. urban). Error bars represent one standard error of the mean.

Figure 6 displays the observed means for reaction time and accuracy across settings (nature vs. urban) and sessions.

### 3.3 Relationship between ERN and behavioral performance

Exploratory analyses did not reveal significant relationships between ERN amplitude and behavioral performance. ERN amplitude did not predict mean RT (χ^2^ = 1.29, *p* = 0.25), nor did it interact with session to predict RT (χ^2^ = 0.42, *p* = 0.810). Similarly, there was no main effect of ERN amplitude on mean accuracy (χ^2^ = 0.22, *p* = 0.64), and no interaction with session (χ^2^ = 0.00*, p* > 0.99).

## 4 Discussion

The present study investigated whether brief exposure to nature imagery could modulate error monitoring processes, as measured by the ERN during the Eriksen Flanker Task. We hypothesized that the ERN amplitude would significantly increase after exposure to nature imagery but would remain unchanged following urban imagery exposure, based on ART's proposal that natural environments restore attentional resources, improving executive function. This hypothesis is also built upon findings from LoTemplio et al. (2020) and McDonnell and Strayer (2024b), who demonstrated enhanced ERN amplitudes specifically following real- world nature exposure. However, our findings revealed no significant differences in ERN amplitude between nature and urban imagery at Session 2, suggesting that a 10-min exposure to the static nature imagery used in our study may not be sufficient to elicit the cognitive benefits previously observed with real-world nature exposure (LoTemplio et al., 2020; McDonnell and Strayer, 2024b). Analysis of behavioral measures revealed no significant interaction effects or main effects of setting for either reaction time or accuracy. While a significant main effect was found within mean reaction time across sessions, this likely reflects practice effects from repeated task exposure and does not provide particular insight into a unique effect of environmental images on behavioral reaction time. These findings align with Johnson et al.'s (2021) meta-analysis showing no significant impact of simulated nature on attention restoration and executive function measures. However, the ERN was successfully elicited across all conditions, suggesting successful measurement of error monitoring processes.

Our exploratory analyses found no relationship between ERN amplitude and behavioral measures of reaction time or accuracy. This dissociation aligns with our introduction's rationale for using neurophysiological measures to detect subtle neural changes that may not manifest in overt behavioral performance. Our results demonstrate that neural measures like the ERN reflect aspects of attentional processing distinct from observable behavioral outcomes and reemphasizes that incorporating both neurophysiological and behavioral metrics when studying attention restoration provides converging yet nuanced measures of attentional processes. Our results also further demonstrate the complicated relationships between the ERN and behavioral performance at the between subjects level of analysis (LoTemplio et al., 2023).

The absence of ERN modulation following nature imagery exposure, contrasting with documented effects of real nature exposure (LoTemplio et al., 2020; McDonnell and Strayer, 2024b), suggests several important theoretical and practical implications. ART proposes that natural environments facilitate cognitive recovery by engaging bottom-up attention while allowing top-down attentional systems to rest. Our findings suggest that static visual stimulation alone may be insufficient to improve attention-related cognitive processes, aligning with recent work by Song et al. (2023) showing that auditory nature exposure alone, while effective for stress reduction, does not significantly impact attention. These parallel findings suggest that nature's impact on attention systems may require integration of multiple sensory inputs, or that nature's effects on attention processes may be more subtle than or modulated by its well-documented stress-reduction benefits. This could explain why static nature imagery interventions, which typically engage only one sensory modality (i.e., vision), show inconsistent effects on attention-related outcomes compared to their more reliable effects on stress and mood measures (Spano et al., 2023).

Our null results challenge simplistic interpretations of ART that might assume any nature exposure—regardless of format, duration, or sensory engagement—would yield cognitive benefits. Instead, they suggest a more nuanced approach is needed to understand the specific conditions under which nature exposure facilitates attentional restoration. If Attention Restoration Theory does explain attentional benefits in nature, it is perhaps unsurprising that nature imagery may not, or may inconsistently, benefit attentional control. One facet of this may be the duration and type of nature images that we used while measuring attentional control. Charbonneau et al. (2024) found a nuanced relationship between exposure duration and cognitive benefits, where 10-s exposures to nature images improved attentional control (on the Flanker Deadline task) but not working memory capacity or its memory components. Previous literature supports this, with contradictory findings regarding attentional control, where a 50-min nature walk yielded no attentional network benefits in one study, while just 6 min of viewing nature images produced positive effects in another (for review see Charbonneau et al., 2024). It is possible that our 10-min nature exposure was the incorrect duration of time to observe attentional control benefits as measured by the Erikson Flanker Task. Kaplan (1995) describes four key components that make nature restorative: fascination, extent, compatibility, and being-away. Within nature imagery, it is possible that specific image characteristics relating to these four concepts may drive cognitive benefits for attentional control beyond it just being “nature.” Previous literature has begun to explore this. Charbonneau et al. (2024) cite research demonstrating that greater fascination led to improved memory performance and recognition accuracy for nature images when viewing them for short durations of time. Charbonneau et al. also found that urban images normed as less fascinating than nature and they encourage researchers to explore these concepts more and to investigate other nature image dimensions like mystery, likability, mindfulness, and resilience as they suggest these properties may drive cognitive benefits. This suggests our null results might result from using nature imagery lacking the optimal psychological properties necessary to trigger attentional restoration. Additionally, “extent” or immersiveness may also be related to cognitive benefits. Our 2D nature images presented on computer screens likely lacked the spatial immersion and multi-sensory engagement that real nature or other types of virtual nature environments may provide. Therefore, our nature imagery alone may not have possessed sufficient “extent” or felt immersive enough to evoke the expected benefits to attention restoration.

While these cognitive benefits have been established in real-world nature conditions (McDonnell and Strayer, 2024b; LoTemplio et al., 2020; Berman et al., 2008), our study did not demonstrate the improvements in attentional control using 2D nature imagery. However, real-world nature may be difficult to access for a number of reasons, especially for groups that may particularly benefit from nature-enhancing executive function improvements, such as older adults who could experience improved executive function for healthy aging. For people who cannot access natural environments due to a variety of factors such as medical limitations, mobility limitations, or incarceration, experiencing nature in a virtual format—including nature imagery—could be a crucial way to facilitate access to nature's health and wellbeing benefits (LoTemplio et al., 2023b; Masters et al., 2022). Therefore, understanding how virtual nature might replicate these same benefits offers significant practical implications for developing accessible interventions.

One interesting medium that researchers are currently exploring is virtual reality (VR) nature. While little research has examined the effect of VR nature on attention, some work has shown that VR environments that include nature lead to improved performance on a Backwards Digit Span task compared to other VR environments (Mostajeran et al., 2021, 2023). Digital representations of nature include pictures, videos, sounds, and simulations, but virtual reality headsets promote nature engagement in a uniquely immersive way (Browning et al., 2023). This increased immersion may be critical for approximating the multisensory experience of real nature. An important avenue for future research would be to examine the effect of multisensory VR nature experiences on neurophysiological markers like ERN amplitude, combining visual, auditory, and potentially other sensory inputs to more closely approximate real-world nature exposure.

These findings, while showing no significant effect of nature imagery on ERN amplitude, should not be interpreted as a broad summation of virtual nature's potential for eliciting attention restoration. Rather, they represent one piece of a complex puzzle regarding how various aspects of nature exposure influence various components of attention and cognitive function. The seemingly contradictory findings in previous literature—such as the varied effects on alpha wave activity observed by Ulrich (1983), Chang et al. (2008), Grassini et al. (2019), and Hopman et al. (2020)—highlight the nuances in our understanding of nature exposure and cognitive processing. Our study's focus on the ERN component and the Flanker task represents just one way of conceptualizing and measuring attention restoration. The lack of significant results in this specific paradigm and design may not indicate that virtual nature will not elicit responses broadly, but rather that there is a need to explore multiple conceptualizations of attention and cognitive function across different attention paradigms in order to build a more comprehensive understanding of how virtual nature influences different aspects of cognitive function.

Additional measurements of attention restoration and cognitive changes in nature vs. virtual nature are beneficial to the study of attention restoration and nature's cognitive benefits. For example, McDonnell and colleagues demonstrated that power in the frontal midline theta (FMθ) frequency band is a robust marker for neurological differences in effortful engagement of executive attention between individuals on nature vs. urban walks. Their 40-min comparison suggested that urban environments are more attentionally straining than natural environments, and these differences could not be attributed to exercise alone (McDonnell and Strayer, 2024a). Additionally, ERPs that measure different aspects of executive function, such as the P300 which may measure the alerting network, found increased alerting after both low-intensity walks in nature and urban interventions, potentially attributed to the exercise effect (McDonnell and Strayer, 2024b). These types of additional measures and conceptualizations of nature could be assessed through virtual nature interventions to build understanding and provide insights into how nature imagery influences attention and cognitive control.

### 4.1 Limitations and future directions

Several important limitations should be considered when interpreting these findings. Although all models were evaluated for key statistical assumptions, some degree of residual non-normality and mild heteroscedasticity persisted—most notably in the accuracy models due to ceiling effects. While various transformations failed to fully resolve these issues, linear mixed-effects models demonstrate robust performance despite such violations, particularly when appropriate random effects structures are implemented and sample sizes are sufficient. Nevertheless, we acknowledge these statistical constraints as a limitation and exercise additional caution when interpreting results. Additionally, the study was originally conceptualized as a dose-response comparison study for the real-world data collected in nature (LoTemplio et al., 2020). However, the COVID-19 pandemic paused data collection on this project for over a year. Therefore, we had to start data collection again with this project as a standalone study. The design would be stronger if the ERN was extracted before and after participants were randomly assigned to either nature imagery or urban imagery, and future work should directly examine this. Also, this study's design could also be strengthened by implementing a within study design directly comparing a nature imagery condition with a comparable real-world nature condition. Our findings' generalizability is further limited by our sample characteristics, which consisted of healthy, young to middle-aged, mostly White adults. Additionally, the nature imagery used in this study may not have been sufficiently immersive to replicate the multisensory experience of real natural environments The specific behavioral measures that we assessed may be a potential limitation. Our study focused exclusively on performance metrics of the Erikson Flanker Task; however, attentional control may be assessed through other behavioral measures (Charbonneau et al., 2024). We did not assess participants' individual previous experiences with nature or outdoor environments, their affinity for it, or their upbringing. However, this may be an important consideration for future directions, as previous research suggests that environmental preferences and familiarity may impact perceived benefits, including perceived restorativeness (Berto et al., 2018). Individual differences in how people respond to nature can influence both their visual preferences for natural settings and significantly impact their health-related outcomes (for review, see Hartig et al., 2011).

Exploring complementary neurocognitive measures could enhance our understanding of attention restoration mechanisms. Recent research investigating reward sensitivity during nature exposure offers valuable insights when considered alongside error monitoring processes. McDonnell et al. (2025), who examined the Reward Positivity (RewP) component—a neural marker of reward sensitivity—during nature exposure. Their four-day immersion study revealed reduced RewP amplitude in natural settings, suggesting diminished sensitivity to extrinsic rewards when immersed in nature. The researchers attributed this to a fundamental shift in cognitive priorities from external rewards (typical in urban environments) to intrinsic rewards associated with nature experiences. Notably, when participants merely viewed nature imagery rather than experiencing immersive natural environments, no significant RewP reduction occurred. This differential impact of real vs. virtual nature parallels our current findings, suggesting consistent patterns across multiple cognitive domains. These converging lines of evidence highlight the importance of examining various presentation methods in future nature-based cognitive research.

Future research in this domain should focus on several key areas to advance our understanding of attention restoration in virtual nature of all types. A primary direction should be the systematic manipulation of distinct aspects of virtual nature exposure to identify which aspects of nature are necessary for attention restoration and what duration of nature exposure is ideal to elicit these benefits. By allowing researchers to precisely control and manipulate environmental variables while maintaining high ecological validity, immersive VR headsets could help isolate the specific characteristics of natural environments that drive attention restoration both for virtual and real-world. Studies could systematically vary factors such as the type of environment (ex., mountains, deserts, urban greenspaces), the presence and type of ambient nature sounds, and the duration of nature immersion. These manipulations could also allow for the improved understanding of the role of incorporating multiple sensory modalities of nature on attention restoration and of the role of dose or duration of nature imagery that might produce effects more similar to real nature exposure. This knowledge would be particularly valuable for developing interventions for populations with limited access to natural environments, potentially allowing more people to benefit from nature's cognitive enhancement effects while still accommodating their physical or situational constraints. Individual and cultural differences' impact on nature preferences and attention restoration should also be explored. Furthermore, studies should employ multiple conceptualizations of attention and cognitive function across different attention paradigms to build a more comprehensive understanding of how virtual nature influences various aspects of cognitive restoration. Additionally, future studies may focus on alternative behavioral measures of attention such the Stroop task and the executive portion of the Attention Network Task (Charbonneau et al., 2024).

## 5 Conclusion

Our findings contribute to the ongoing discourse regarding the efficacy of nature imagery on attention restoration. While some studies have found comparable benefits between nature imagery and real nature exposure (Berman et al., 2008; Crossan and Salmoni, 2021), our neurophysiological data suggests that brief nature imagery may not replicate the effects of real nature exposure on error monitoring and cognitive control processes. This discrepancy highlights the need for more sensitive neurophysiological measurements, including expanded EEG paradigms, as well as different conceptualizations of attention that may better capture the nuanced ways virtual nature, including nature imagery, affects cognitive processing.

These findings have important practical implications for the development and implementation of nature-based interventions. While virtual nature offers practical advantages in terms of accessibility and scalability, our results suggest that the simple 2D nature imagery used in our paradigm may not be an effective substitute for real nature exposure for attention restoration. Understanding the specific components and duration of nature exposure that drive cognitive enhancement will be crucial for developing more effective approaches to virtual nature interventions, potentially incorporating multiple sensory modalities or more immersive technologies such as virtual reality, while also considering individual or group differences. This is particularly important given that virtual nature experiences may be especially valuable for individuals who face barriers to accessing real natural environments, such as those with mobility limitations, those living in urban areas with limited green space, or those facing other physical or socioeconomic constraints.

## Data Availability

The original contributions presented in the study are publicly available. This data can be found here: https://osf.io/u8kd5/?view_only=1533429827ae4606838438de4b870120.
